# Pharmacological activation of SIRT6 triggers lethal autophagy in human cancer cells

**DOI:** 10.1038/s41419-018-1065-0

**Published:** 2018-09-24

**Authors:** Sara Iachettini, Daniela Trisciuoglio, Dante Rotili, Alessia Lucidi, Erica Salvati, Pasquale Zizza, Luca Di Leo, Donatella Del Bufalo, Maria Rosa Ciriolo, Carlo Leonetti, Clemens Steegborn, Antonello Mai, Angela Rizzo, Annamaria Biroccio

**Affiliations:** 1 Oncogenomic and Epigenetic Unit, IRCCS-Regina Elena National Cancer Institute, Via Elio Chianesi 53, 00144 Rome, Italy; 2 Preclinical Models and New Therapeutic Agents Unit, IRCCS-Regina Elena National Cancer Institute, Via Elio Chianesi 53, 00144 Rome, Italy; 3 Institute of Molecular Biology and Pathology, CNR National Research Council, Via degli Apuli 4, 00185 Rome, Italy; 4 Department of Drug Chemistry and Technologies, Sapienza University, Piazzale Aldo Moro 5, 00185 Rome, Italy; 5 Department of Biology, University of Rome “Tor Vergata”, Via della Ricerca Scientifica 1, 00133 Rome, Italy; 6 SAFU, IRCCS-Regina Elena National Cancer Institute, Via Elio Chianesi 53, 00144 Rome, Italy; 7 Department of Biochemistry, University of Bayreuth, 95440 Bayreuth, Germany

## Abstract

Sirtuin 6 (SIRT6) is a member of the NAD^+^-dependent class III deacetylase sirtuin family, which plays a key role in cancer by controlling transcription, genome stability, telomere integrity, DNA repair, and autophagy. Here we analyzed the molecular and biological effects of UBCS039, the first synthetic SIRT6 activator. Our data demonstrated that UBCS039 induced a time-dependent activation of autophagy in several human tumor cell lines, as evaluated by increased content of the lipidated form of LC3B by western blot and of autophagosomal puncta by microscopy analysis of GFP-LC3. UBCS039-mediated activation of autophagy was strictly dependent on SIRT6 deacetylating activity since the catalytic mutant H133Y failed to activate autophagy. At the molecular level, SIRT6-mediated autophagy was triggered by an increase of ROS levels, which, in turn, resulted in the activation of the AMPK-ULK1-mTOR signaling pathway. Interestingly, antioxidants were able to completely counteract UBCS039-induced autophagy, suggesting that ROS burst had a key role in upstream events leading to autophagy commitment. Finally, sustained activation of SIRT6 resulted in autophagy-related cell death, a process that was markedly attenuated using either a pan caspases inhibitor (zVAD-fmk) or an autophagy inhibitor (CQ). Overall, our results identified UBCS039 as an efficient SIRT6 activator, thereby providing a proof of principle that modulation of the enzyme can influence therapeutic strategy by enhancing autophagy-dependent cell death.

## Introduction

Sirtuins are histone deacetylase enzymes that use nicotinamide adenine dinucleotide (NAD^+^) as a co-substrate for their enzymatic activities. They are mainly involved in regulation of cell stress response and metabolism, thus playing key roles in normal and cancer cells^1^. Among the components of mammalian sirtuin family, SIRT6 deacetylates the histone H3 on acetylated K9, K56^2,3^, and the more recently identified K18 and K27 residues^4,5^, causing the repression of many genes involved in inflammation, aging, genome stability, metabolic pathways, and telomere integrity^2,6–8^. Moreover, many functions of SIRT6 are linked to its ability to deacetylate and catalyze mono-ADP-ribosylation of non-histone proteins, including transcription factors and other proteins involved in DNA damage response, inflammation, and immune response activation^7,9–13^. Due to its active role in several important biological processes, SIRT6 dysregulation has been implicated in the onset of several pathologies^14,15^. In cancer, the role of SIRT6 is controversial^14^. In some tumors, SIRT6 acts as a tumor suppressor; indeed, SIRT6 expression has been found downregulated in many human tumors (i.e. colorectal, breast, ovarian, hepatocellular, lung, and pancreatic tumors) and its downregulation is associated with poor prognosis^16–18^. Consistent with these results, loss of SIRT6 leads to tumor formation and maintenance^8^ and ectopic expression of SIRT6 inhibits cancer stem cell proliferation^19,20^. In other tumors (i.e. skin cancer, hepatocarcinoma, multiple myeloma, and acute myeloid leukemia), SIRT6 can act as a tumor promoter and its overexpression has been associated to poor outcomes^21–23^. Recent evidences reveal a role of sirtuins, including SIRT6, in autophagy of several biological systems^24–28^. In normal cells, SIRT6-mediated induction of autophagy is involved in oxidative stress-induced neuronal damage^29^, bronchial epithelial cell senescence^30^, cardiac hypertrophy^26^, and monocyte differentiation^31^. In cancer, the role of SIRT6 in autophagic processes has been poorly investigated. In particular, in esophageal cancer cells, SIRT6 induces autophagy by activating ULK1 and inhibiting mTOR pathway^21^, while in melanoma it differently affects tumor growth of primary and metastatic tumors in an autophagy-dependent manner via the IGF-AKT signaling pathway^32^.

Autophagy is a highly conserved multistep process that is fundamental to maintain cellular homeostasis. During this process, unfolded proteins or damaged organelles are engulfed by double-membrane autophagosomes and are delivered to lysosomes for degradation^33^. Defects in autophagy have been associated with susceptibility to genomic damage, metabolic stress, and, importantly, tumorigenesis^34^. In recent years, an increasing number of studies have provided a plethora of conflicting results about the role of autophagy in cancer biology. Indeed, in cancer cells, autophagy has a dual role, acting as a mechanism of tumor suppression or as an adaptive stress response to maintain tumor cell survival. Moreover, there is a functional crosstalk between autophagy and apoptosis, and either increased or blocked autophagic flux may induce apoptotic cell death in various conditions^35^. To date, there are only few modulators of the autophagic pathway that have shown promising pharmacological value^36^.

In this contest, exploiting the possibility to act on the autophagic process, through a direct modulation of SIRT6, could be of fundamental importance representing a novel avenue in cancer therapy.

UBCS039 has been recently described as the first synthetic activator of SIRT6^37^. Here we explored the molecular and biological effects of this compound in cancer cell lines of different origin, including non-small cell lung, colon and epithelial cervix carcinoma, and fibrosarcoma, clearly demonstrating that pharmacological SIRT6 activation triggers an autophagy-related cell death.

## Materials and methods

### Cells and culture conditions

H1299 human non-small cell lung cancer, HT1080 human fibrosarcoma, HCT116 human colon, and HeLa human epithelial cervix carcinoma cell lines were purchased from American Type Culture Collection. HeLa and HCT116 were cultured in Dulbecco’s modified Eagle’s medium (DMEM; Euroclone, ECM0728L), H1299 and HT1080 in Roswell Park Memorial Institute medium (RPMI 1640; Gibco, 21875–034), both supplemented with 10% fetal bovine serum. The indicated cell lines were grown in a CO_2_ humidified incubator at 37 °C. HT1080 and H1299 cells were stably transfected with EGFP-LC3B or mRFP-EGFP-LC3B fusion proteins as previously reported^38,39^ and cultured in the presence of 800 μg/ml of geneticin (G418 disulfate salt, Sigma-Aldrich, A1720).

### Reagents

SIRT6 activator UBCS039 and the regioisomer negative control UBCS060 were synthesized as previously reported^37^ and freshly dissolved in dimethylsulfoxide (DMSO) before any experiment. Chloroquine diphosphate (CQ, Sigma-Aldrich, C6628) was freshly dissolved in water, *N*-acetyl-l-cysteine was freshly dissolved in 12% NaOH 5 M (*N*-acetyl-l-cysteine (NAC), Sigma-Aldrich, A7250). 3-methyladenine (3-MA, Enzo Life Science, BML-AP502–0025), Trolox (Sigma-Aldrich, 238813), and zVAD-fmk (Calbiochem, 627610) were dissolved in DMSO. For all the experiments, cells were treated with DMSO as a control.

### Western blot

After treatments, cells were collected and lysed in a buffer containing 50 mM Tris-HCl (pH 7.5), 5 mM EDTA, 250 mM NaCl, and 0.1% Triton, and completed with protease (ThermoScientific, A32953) and phosphatase inhibitors (ThermoScientific, 88667). Total protein extracts were fractionated by SDS-polyacrylamide gel electrophoresis, transferred to a nitrocellulose filter, and subjected to immunoblot assay. Following primary antibodies were used: H3K9Ac (Millipore, 07–352); H3K56Ac (Abcam, ab76307); SIRT6 (Novus Biologicals, NBP1-30101); LC3B (Sigma-Aldrich, L7543); total AMP-activated protein kinase (AMPK; Cell Signaling, 2532) and phospho AMPK (Thr172; Cell Signaling, 2535); total (Calbiochem, ST1521) and phospho ULK1 (Ser555 and Ser757; Cell Signaling, 5869 and 6888 respectively); total mTOR (Cell Signaling, 4517) and phospho mTOR (ser2448; Cell Signaling, 2971); Beclin-1 (Cell Signaling, 3738); ATG5 (Cell Signaling, 2630); PARP1 (BD Pharmingen, 551025); β-actin (Sigma, A5441); HSP72/73 (Calbiochem, 386032); and H3 (Abcam, ab1791). Following secondary antibodies were used: Goat anti-mouse or anti-rabbit immunoglobulin G (IgG)-horseradish peroxidase conjugated antibodies (Biorad, 1706516 and 1706515 respectively). Densitometry was performed using ImageJ software.

### Immunofluorescence microscopy

HT1080 and H1299 stably expressing EGFP-LC3B or mRFP-EGFP-LC3B fusion proteins were grown on glass coverslips, treated with UBCS039, and fixed in 2% formaldehyde in phosphate-buffered saline (PBS) 1× for 10 min at room temperature. Autophagosome structure formation was detected observing LC3B puncta in EGFP-LC3B-expressing cells, while autophagosome maturation was assessed analyzing yellow- and red-fluorescence structures in mRFP-EGFP-LC3B (ptf-LC3)-expressing cells, indicating an accumulation of autophagosomes and autolysosomes, respectively. Evaluation of autophagosome structure formation and maturation was performed independently and in blinded manner by two investigators counting at least 250 cells for experimental conditions, cells with more than 10 puncta were considered autophagy positive. Co-localization experiments were performed in H1299 EGFP-LC3B cells stained with primary antibody against LAMP-2 (H4B4; Santa Cruz Biotechnology, sc-18822). Anti-Rabbit IgG (Alexa Fluor 555 Conjugate, Cell Signaling, 4413) was used as a secondary antibody. Preparations were analyzed under an Olympus AX70 microscope using a ×100/1.35 numerical aperture objective. Images were acquired using a TCH-1.4ICE camera (Tucsen, Fujian, China) controlled by ISCapture and processed using Adobe PhotoShop software (Adobe Inc., Burlington, NJ, USA).

### ATP levels

HeLa cells were treated with 100 µM UBCS039 for 24 h, detached, and disrupted by three cycles of freezing-thawing in dry ice with ethanol in a buffer containing 100 mM Tris-HCl, pH 7.75, and 4 mM EDTA. Lysates were incubated for 2 min at 100 °C and ATP levels measured by the ATP Bioluminescence Assay Kit CLS II (Roche Applied Science, 11 699 695 001), according to the manufacturer instructions, using a microplate luminometer (PerkinElmer, Waltham, Massachusetts, U.S.). Obtained values were normalized on total protein amount.

### Reactive oxygen species production

Cells were treated with 100 µM UBCS039 for different times, harvested, washed in PBS 1× and stained for 30 min at 37 °C with dihydroethidium 50 µM (ThermoScientific D1168) dissolved in DMEM without fetal bovine serum. Cells treated with H_2_O_2_ 5 mM for 30 min were used as a positive control. About 15 000 events were acquired and analyzed by using BD Accuri™ C6 flow cytometer (BD Biosciences, San Diego, CA, U.S.) and gated using forward scatter and side scatter to exclude cell debris.

### Cell proliferation and cell cycle analysis

To test the effect of UBCS039 on cell proliferation, 5 × 10^4^ cells were plated in 60 mm Petri dish. After 24 h from seeding, exponentially growing cells were treated with the compound at the indicated concentrations for 24, 48, and 72 h. Cells were collected and cell number was determined by direct counting with hemocytometer. Progression of cells through cell cycle phases and apoptosis were analyzed in both floating and adherent cells by propidium iodide (PI, BD Biosciences, 556463) and AnnexinV-fluorescein isothiocyanate (BD Biosciences, 556420)/PI staining respectively, as previously described^39^. All flow cytometric analyses were performed by using BD Accuri™ C6 flow cytometer (BD Biosciences, San Diego, CA, U.S.).

### Statistics

Experiments were replicated three times and the data were expressed as mean ± standard deviation or mean ± standard error. Differences between groups were analyzed with a two-sided paired or unpaired *t* test and they were considered to be statistically significant for *P < 0.05; **P < 0.01; ***P < 0.001; ****P < 0.0001.

## Results

### UBCS039 induces deacetylation of SIRT6-targeted histone H3 sites in human cancer cells

**Fig. 1:**
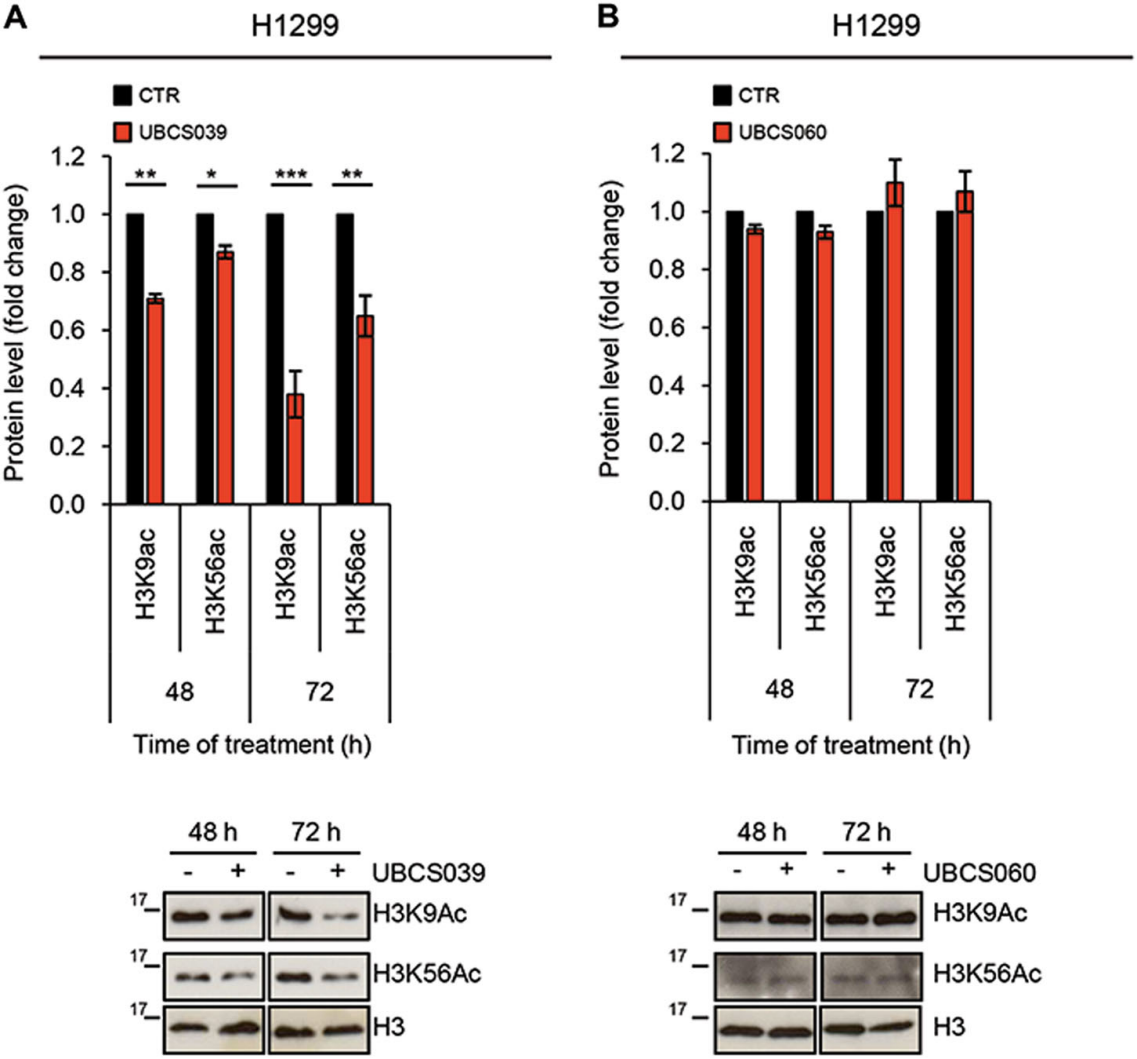
UBCS039 induces deacetylation of SIRT6-targeted histone H3 sites in human H1299 cells. **a**, **b** Western blot analysis of acetylation levels of histone H3K9 and H3K56 in H1299 human non-small cell lung cancer cell line treated with 75 µM of SIRT6 activator UBCS039 (**a**) or of the chemical analog UBCS060 (**b**) for the indicated times. H3 acetylation levels were quantified by densitometric analyses using ImageJ software and the relative levels of histone modifications were expressed in the histograms as fold changes of treated versus untreated samples, after H3 normalization. The results represent mean ± SD of three independent experiments. *P < 0.05; **P < 0.01; ***P < 0.001

UBCS039 is a newly synthesized pyrrolo[1,2-a]quinoxaline derivative, recently described as the first synthetic activator of the NAD^+^-dependent protein lysine deacetylase SIRT6^37^. Here we evaluated the effect of UBCS039 treatment on SIRT6 by analyzing H3 acetylation levels on K9 and K56 residues (H3K9 and H3K56), two typical targets of SIRT6 activity. As shown by western blot analysis in Fig. 1a, UBCS039 induced a time-dependent deacetylation of histone H3K9 in H1299 non-small cell lung cancer. Pharmacological activation of SIRT6 by UBCS039 was obtained in several cell lines regardless of tumor histotype and without affecting SIRT6 protein expression levels (Supplementary 1A, B). In particular deacetylation of histone H3K9 was found in HeLa epithelial cervix carcinoma and HCT116 colon carcinoma lines, while no effect was observed in HT1080 human fibrosarcoma cell line expressing a higher basal level of H3K9 histone variant (Supplementary 1A). UBCS039 also triggered deacetylation of histone H3K56 in HT1080, while this effect is not appreciable in HeLa and HCT116 cell lines, showing no detectable levels of this histone post-translational modification.

Interestingly, UBCS060, a pyrrolo[1,2-a]quinoxaline regioisomer of UBCS039, showing very low SIRT6 affinity and no effects on SIRT6-dependent deacetylation in vitro^37^ failed to stimulate deacetylation of H3K9 and H3K56 in H1299 cells (Fig. 1b). Moreover, knockdown of SIRT6, by increasing in a time dependent manner H3K9 acetylation levels, exerted opposite effects compared to UBCS039 treatment (Supplementary 1C). Altogether, the results demonstrated that UBCS039 is a specific activator of SIRT6 in several human tumor models.

### UBCS039 leads to autophagosome accumulation in human cancer cells

**Fig. 2:**
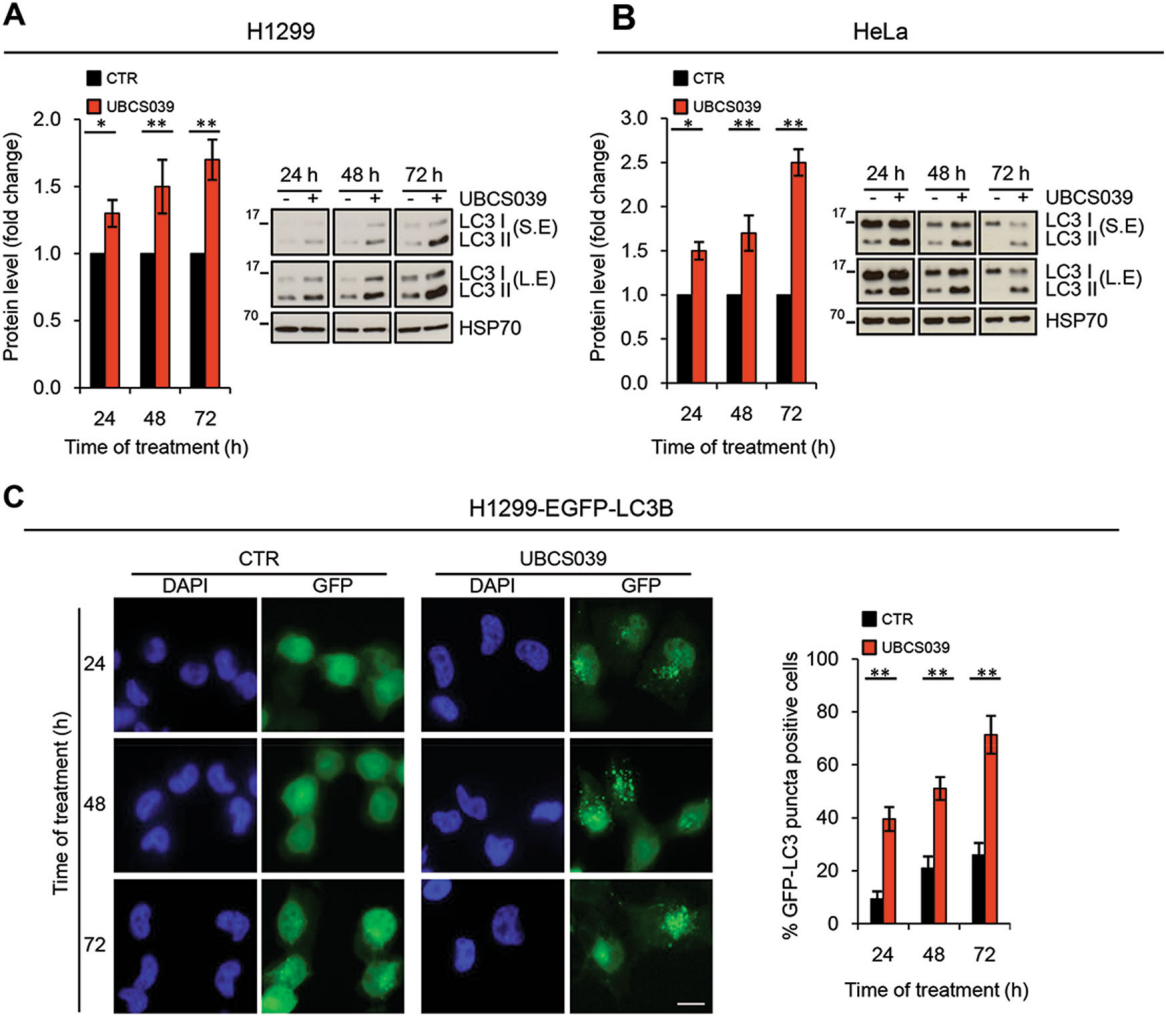
UBCS039 induces autophagosome accumulation. **a**, **b** Western blot analysis of microtubule-associated protein 1 form II (LC3B-II) protein level in H1299 (**a**) and HeLa (**b**) cells treated with 75 µM UBCS039 for the indicated times. LC3B-II levels were quantified by densitometric analyses using ImageJ software and the relative levels of LC3B-II were expressed in the histograms as fold changes of treated versus untreated samples, after HSP70 normalization. *P < 0.05; **P < 0.01. S.E. short exposure, L.E. long exposure. **c** Representative images and relative quantification of fluorescence microscopic analysis of LC3B puncta positive cells in H1299 EGFP-LC3B treated as indicated in **a**. Olympus AX70 microscope, ×100 magnification. Scale bar indicates 10 µm. The results represent the mean ± SEM of three independent experiments. **P < 0.01

Recent works demonstrate the involvement of SIRT6 in autophagy in different cellular contexts, including cancer^21,26,29–31^, but specific SIRT6 activators have not been described yet. Here we investigated the effect of UBCS039 on autophagy signaling and progression. As depicted in Fig. 2, UBCS039 enhanced, in a time-dependent manner, LC3B conversion from LC3B form I (18 kDa) to an autophagosome-associating form, LC3 form II (16 kDa) in both human H1299 and HeLa cell lines (Fig. 2a, b). By using H1299 cells stably transfected with EGFP-LC3B fusion protein, we also found a time-dependent enhancement of LC3B punctate structure formation (Fig. 2c), indicative of autophagosome accumulation. UBCS039-induced autophagosome accumulation was also observed in HT1080 fibrosarcoma cells stably transfected with EGFP-LC3B fusion protein, thus indicating a general effect on autophagy upon activation of SIRT6 (Supplementary 2).

**Fig. 3:**
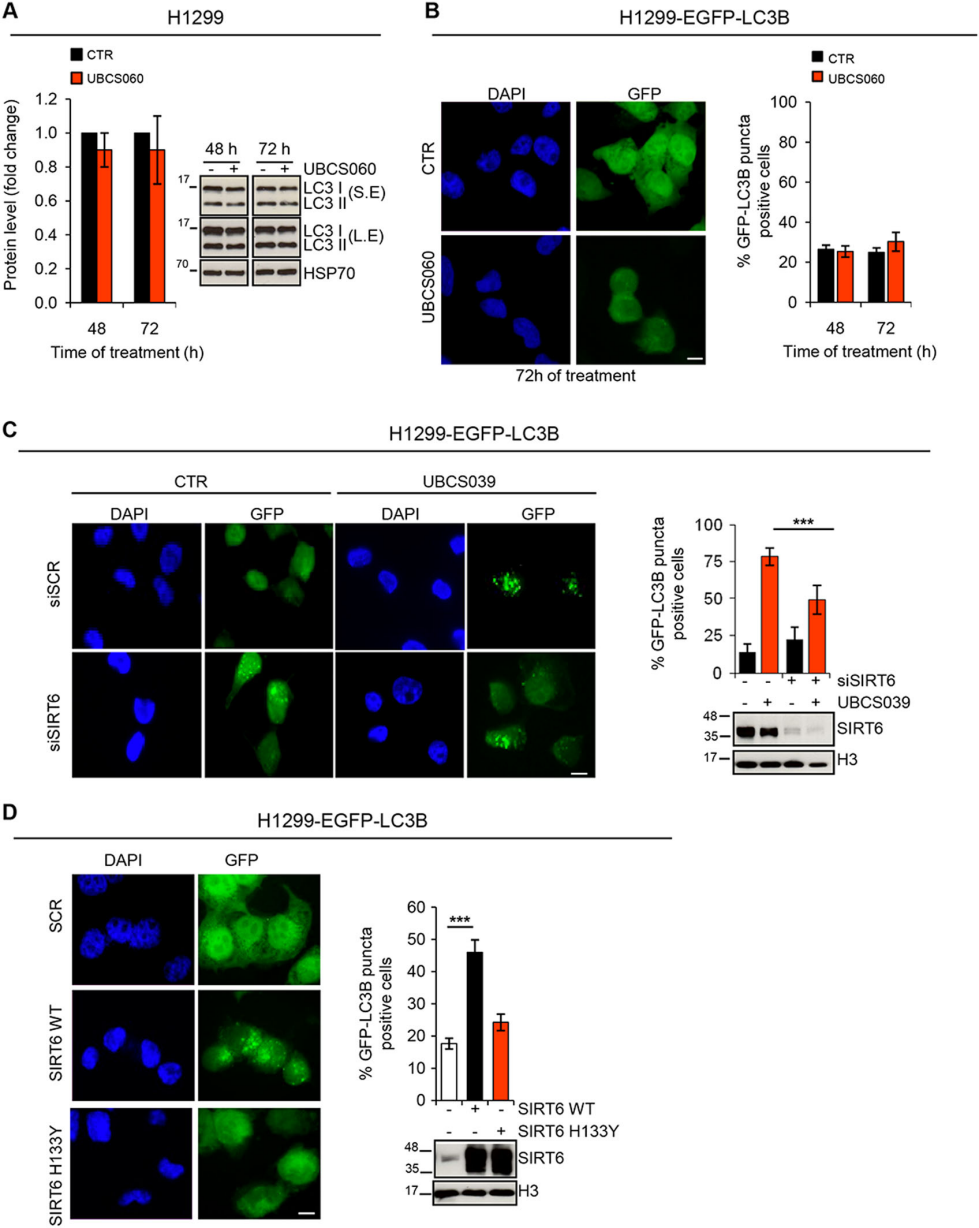
UBCS060, a chemical inactive analog of UBCS039, fails to deaceylate H3K9 and H3K56 and does not induce autophagosome accumulation. **a** Western blot analysis of LC3B-II protein level in H1299 cells treated with 75 µM UBCS060 for the indicated times. LC3B-II levels were quantified by densitometric analyses using ImageJ software and the relative levels of LC3B-II were expressed in the histograms as fold changes of treated versus untreated samples, after HSP70 normalization. S.E. short exposure, L.E. long exposure. **b** Representative images and relative quantification of fluorescence microscopic analysis of LC3B puncta positive cells in H1299 EGFP-LC3B treated as indicated in **a**. **c** Representative images and relative quantification of fluorescence microscopic analysis of LC3B puncta positive cells in H1299 EGFP-LC3B transiently silenced for SIRT6 and treated with UBCS039 75 µM for 72 h. Western blot analysis of SIRT6 expression levels. H3 was used as an internal loading control. **d** Representative images and relative quantification of fluorescence microscopic analysis of LC3B puncta positive cells in H1299 EGFP-LC3B transiently transfected with the wild-type (WT) or with the catalytically inactive form of SIRT6 protein (H133Y) for 48 h. Western blot analysis of SIRT6 expression levels. H3 was used as an internal loading control. **b**–**d** Olympus AX70 microscope, ×100 magnification. Scale bar indicates 10 µm. The results represent mean ± SD of three independent experiments. ***P < 0.001

Consistent with the lack of deacetylation activity, H1299 cells exposed to the chemical analog UBCS060 did show neither significant changing in the accumulation of lipidated LC3B protein nor differences in the percentage of LC3B puncta positive cells (Fig. 3a, b). More interesting, a significant difference in terms of UBCS039-induced accumulation of LC3B puncta positive cells was observed in H1299 SIRT6-depleted cells when compared to H1299 cells transfected with control small interfering RNA (Fig. 3c). Finally, UBCS039-induced autophagic vesicle accumulation was recapitulated by overexpressing the wild-type form of SIRT6 enzyme, but not with the H133Y catalytically inactive mutant (Fig. 3d), clearly demonstrating that deacetylase activity of SIRT6 was responsible for autophagosome accumulation.

### UBCS039 induces autophagic flux in human cancer cells

**Fig. 4:**
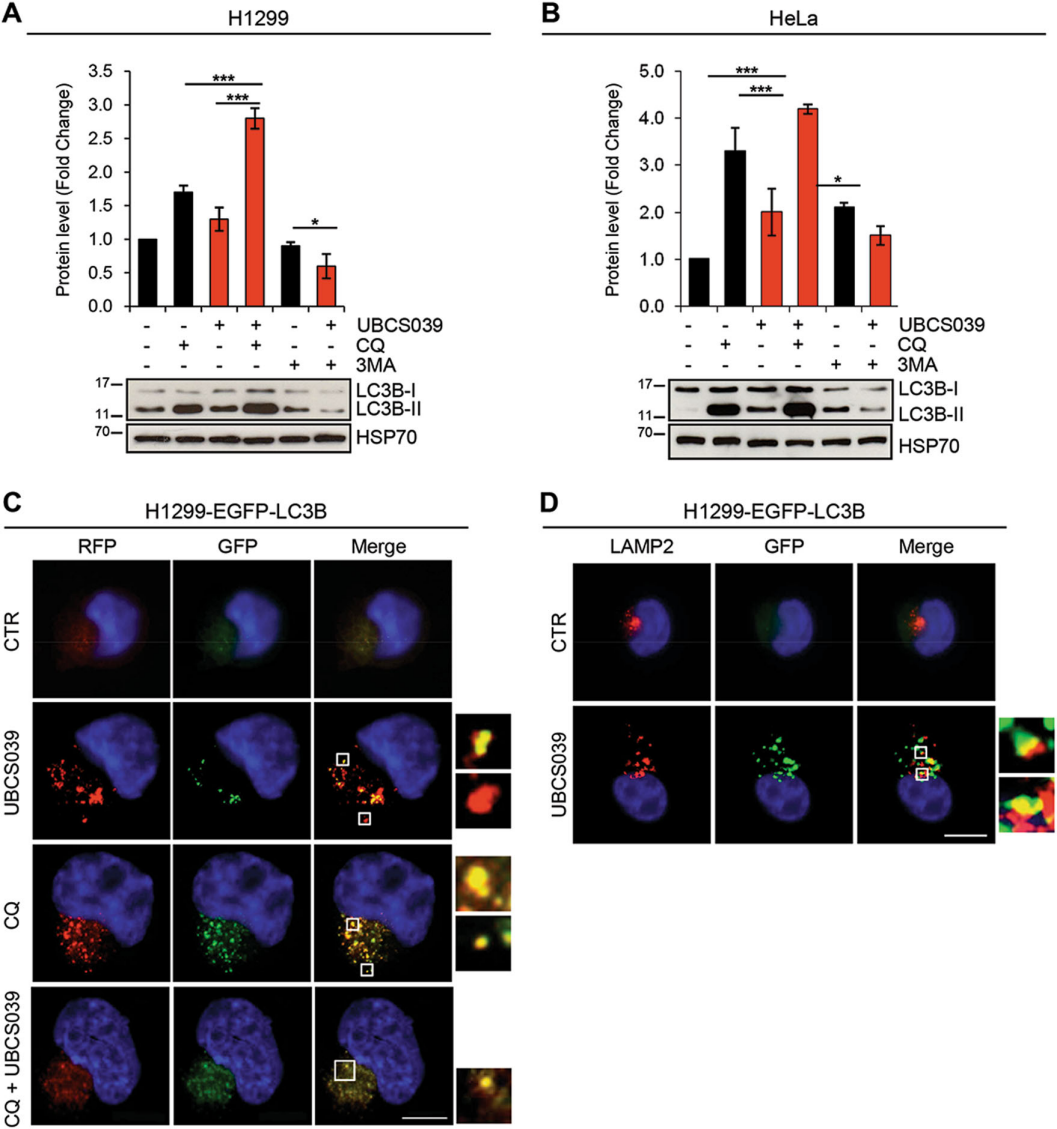
UBCS039 promotes autophagic flux in human cancer cells. **a**, **b** Western blot analysis of LC3B-II protein level in H1299 (**a**) and HeLa (**b**) cell lines treated with 75 µM UBCS039 in combination or not with 3-MA (1 mM) or CQ (25 µM) for 24 h. LC3B-II levels were quantified by densitometric analyses using ImageJ software and the relative levels of LC3B-II were expressed in the histograms as fold changes of treated versus untreated samples, after HSP70 normalization. Results represent mean ± SD of three independent experiments. *P < 0.05; ***P < 0.001. **c** H1299-ptf-LC3 cells were treated with 75 µM UBCS039 for 72 h or with 25 µM CQ for 24 h alone or in combination. Representative images of fluorescence microscopy analysis of yellow- and red-fluorescence EGFP-LC3B punctate vesicular structures, indicative of autophagosomes and autolysosomes accumulation, respectively. **d** Representative images of co-immunofluorescence analysis of lysosomal marker LAMP-2 and EGFP-LC3B punctate vesicular structures in H1299 cells expressing EGFP-LC3 treated or not with 75 µM UBCS039 for 72 h. **c**, **d** Olympus AX70 microscope, ×100 magnification. Scale bar indicates 10 µm

Autophagosome accumulation can be indicative of an increased de novo autophagosome biosynthesis or of an autophagy inhibition^40^. To study the effect of UBCS039 on autophagic flux H1299 and HeLa cell lines were treated with UBCS039 for 24 h in combination or not with the late-stage inhibitor CQ. As expected, CQ treatment caused an increase of LC3B conversion from form I to II, which reflects the basal level of autophagy of these cells (Fig. 4a, b). This effect was more evident when cells were exposed to the combination of UBCS039 and CQ, demonstrating that pharmacological activation of SIRT6 can induce a complete autophagic flux in these cells. Notably, autophagy inhibition by the early-stage autophagy inhibitor 3-MA completely blocked the effect of UBCS039 treatment on LC3B form I–II conversion in both H1299 and HeLa cell lines (Fig. 4a, b). These observations were also strengthened by fluorescence microscopy experiments performed in H1299 cells expressing mRFP-EGFP tandem fluorescence-tagged LC3B construct (ptf-LC3; Fig. 4c). As expected, the autophagic inhibitor CQ alone or in combination with UBCS039 caused an extended increase in the yellow-fluorescence structures, indicative of incomplete impaired autophagosome maturation in lysosomes (autolysosomes). On the contrary, following UBCS039 treatment an accumulation of both yellow- and red-fluorescence structures was observed, indicating an accumulation of both autophagosomes and autolysosomes, respectively. In line with this evidence, the lysosomal marker LAMP-2 co-localized with EGFP-LC3 punctate vesicular structures in H1299 EGFP-LC3 cells treated with UBCS039 (Fig. 4d), confirming that pharmacological activation of SIRT6 promotes the correct fusion between autophagosomes and lysosomes.

### UBCS039 triggers reactive oxygen species accumulation and activates the AMPK/ULK1 signaling pathway

**Fig. 5:**
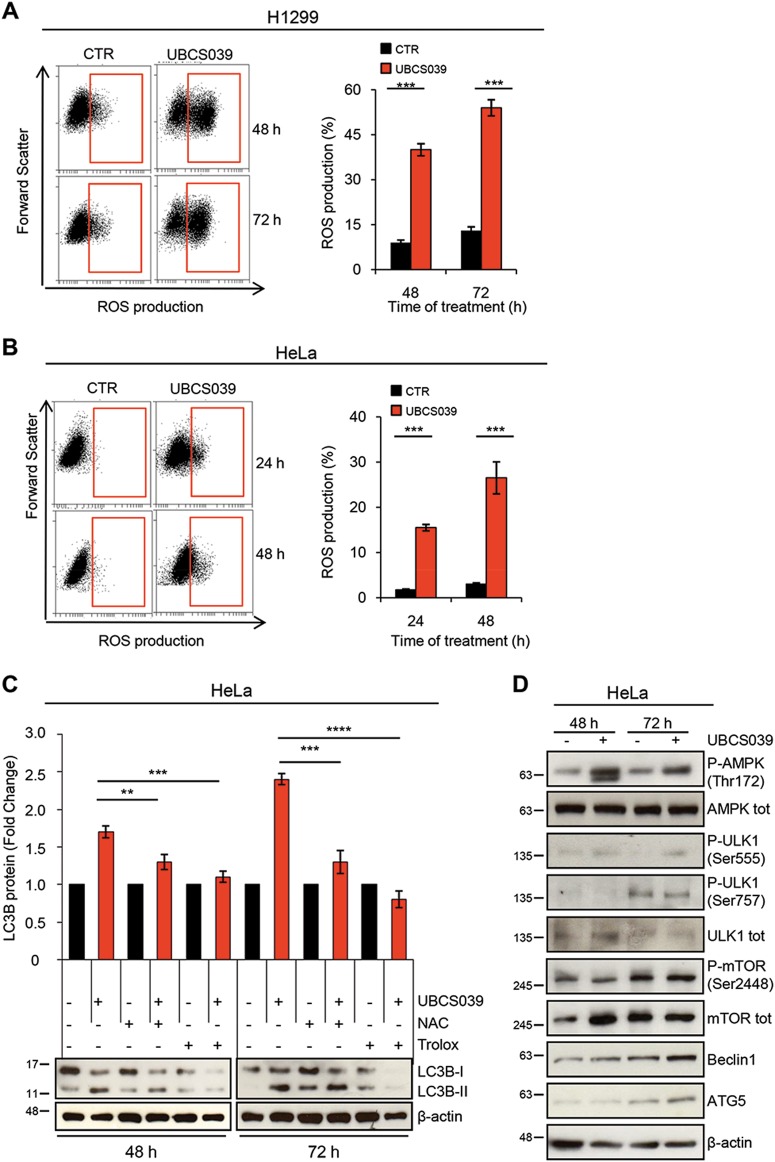
UBCS039 induces autophagy via AMP-activated protein kinase (AMPK) signaling pathway activation. **a**, **b** Representative experiment of flow cytometric analysis of ROS production by DHE staining in H1299 (**a**) and HeLa (**b**) cell lines treated with 100 µM UBCS039 for the indicated times. ROS production was expressed in the histograms as percentage of cells positive for DHE staining. Results represent mean ± SD of three independent experiments. ***P < 0.001. **c** Western blot analysis of LC3B-II protein levels in HeLa cells treated with 100 µM UBCS039 in combination or not with antioxidant compounds NAC (5 mM) and Trolox (200 µM) for the indicated times. LC3B-II levels were quantified by densitometric analyses using ImageJ software and the relative levels of LC3B-II were expressed in the histograms as fold changes of treated versus untreated samples, after β-actin normalization. Results represent mean ± SD of three independent experiments. **P < 0.01; ***P < 0.001; ****P < 0.0001. **d** Western blot analysis of the indicated proteins in HeLa cells treated with 100 µM UBCS039 for the indicated times. Total AMPK, total ULK1, total mTOR, and HSP70 were used as internal loading controls

A very recent study pointed out a protective role of SIRT6 in maintaining cardiac homeostasis, demonstrating that SIRT6 deficiency resulted in decreased oxygen consumption rate and reduced ATP production^41^. Consistently with this study, we demonstrated that UBCS039 treatment was effective in promoting intracellular reactive oxygen species (ROS) accumulation in a time-dependent manner in both H1299 and HeLa cell lines (Fig. 5a, b). An increase in ATP levels was also observed after treatment of HeLa cells with UBSC039 (Supplementary 2). Since a link between ROS and autophagy has been described^42,43^, HeLa cells were treated with UBCS039 in combination with NAC or Trolox antioxidant compounds and LC3B conversion was analyzed. Our results revealed that NAC and, more efficiently, Trolox impaired LC3B conversion to form II, indicating that ROS burst has a key role in the upstream events leading to UBCS039-induced autophagy (Fig. 5c).

Next, we analyzed the impact of UBCS039 on AMPK, a sensor kinase that is activated by changes in ATP levels or oxidative stress, which in turn, can inhibit the mTOR pathway and/or activate the ULK1 protein complex, responsible for the initiation step of autophagy^44^. As shown in Fig. 5d, UBCS039 treatment increased AMPK phosphorylation at Thr172, decreased the mTOR phosphorylation at Ser2448, and concomitantly induced the activation of ULK1. In particular, we found increased ULK1 phosphorylation levels at Ser555 (active ULK1 form) and no alteration at Ser757 (inactive ULK1 form). Moreover, the ULK1-regulated ATG proteins Beclin-1 and ATG5, required for phagophore formation and elongation, mediated the autophagy cascade. Notably, UBCS039 treatment increased AMPK phosphorylation also in H1299 cells, and most remarkably, pretreatment of cells with Trolox partially counteracted the effect of UBCS039 on AMPK signaling pathway (Supplementary 3B). Overall, these results demonstrate that pharmacological activation of SIRT6 induces autophagy by ROS-mediated AMPK/ULK1 pathway activation.

### UBCS039 induces autophagy-associated cell death

**Fig. 6:**
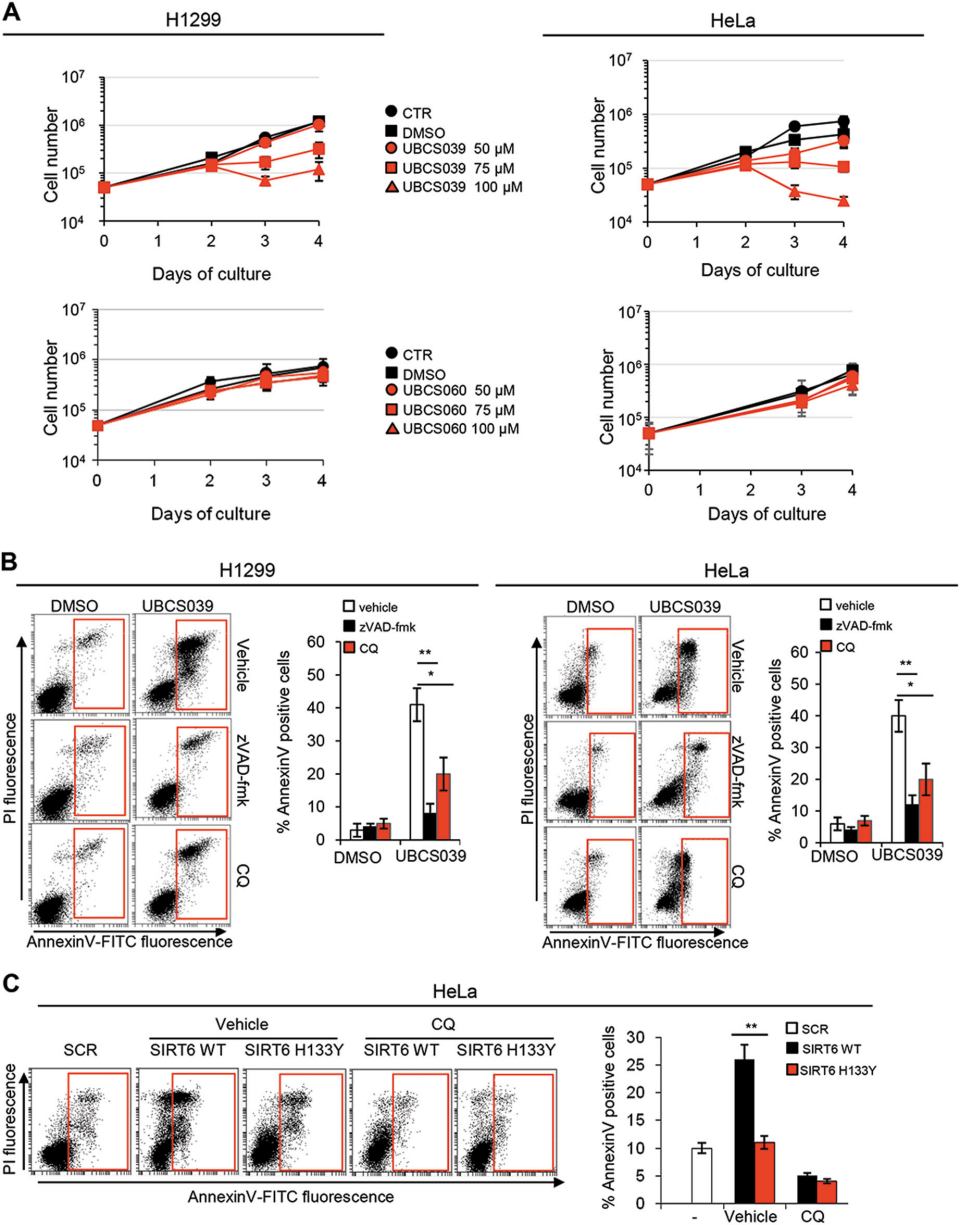
UBCS039 leads to autophagy-mediated apoptosis. **a** In vitro growth curves of H1299 and HeLa cell lines treated or not with UBCS039 or UBCS060 at the indicated doses. In all, 5 × 10^4^ cells were seeded at day 0 and treated at day 1. The figure represents mean ± SD of three independent experiments. **b** Representative experiment of flow cytometric analysis of AnnexinV-positive cells in H1299 and HeLa cell lines treated with 100 µM UBCS039 for 72 h in combination or not with pan-caspase inhibitor zVAD-fmk (50 μM) or the late-stage autophagy inhibitor CQ (25 μM). The results represent mean ± SD of three independent experiments. **c** Representative experiment of flow cytometric analysis of AnnexinV-positive cells in HeLa human epithelial cervix carcinoma cell lines transiently transfected with the empty vector (SCR) and with the wild-type (WT) or the catalytically inactive form of SIRT6 protein (H133Y) for 72 h. The results represent mean ± SD of three independent experiments. *P < 0.05; **P < 0.01

In cancer, it is still unclear if autophagy may promote cell death or cause chemoresistance. Therefore, in order to analyze the consequence of UBCS039-induced autophagy activation, the impact of SIRT6 activator on cell growth was exploited. To this aim, we analyzed the proliferation rate of cancer cells exposed to different doses of the SIRT6 activator UBCS039 or the chemical inactive analog UBCS060. As shown in Fig. 6a, UBCS039 led to a strong decrease of cell proliferation in a dose-dependent manner when compared with control or DMSO-treated cells, starting from day 3 of growth (48 h after treatment) in both H1299 and HeLa cell lines. By contrast, no significant effect on cell proliferation was observed in cells exposed the chemical analog UBCS060 (Fig. 6a). Moreover, UBCS039-induced cell growth inhibition was of general application because it was observed also in other tumor cell lines (Supplementary 4A).

Successively, we investigated if cell growth inhibition was associated with apoptosis induction. Flow cytometric analysis of AnnexinV staining in both H1299 and HeLa cells demonstrated the ability of UBCS039 to induce apoptosis (Fig. 6b). These results were also corroborated by the analysis of sub-G1 peak of cell cycle distribution and by western blot analysis of PARP1 cleaved form (Supplementary 4B–D). Interestingly, exposure of cells to zVAD-fmk, a pan-caspase inhibitor, was able to revert UBCS039 effect on apoptosis (Fig. 6b). Moreover, exposure of cells to the autophagy inhibitor CQ reduced (of about 50%) the apoptotic effect of UBCS039 (Fig. 6b), indicating an involvement of autophagy in UBCS039-mediated apoptosis. Finally, the role of SIRT6 on autophagy-mediated cell death was corroborated by a genetic approach demonstrating that overexpression of wild-type, but not the catalytic inactive form of SIRT6 was able to increase cell death that was reverted by CQ treatment (Fig. 6c). Taken together, these results clearly demonstrate that pharmacological activation of SIRT6 triggers an increase of autophagy that results in apoptosis in different human cancer cells.

## Discussion

SIRT6 involvement in autophagy has recently been reported in different cell contexts, including cancer^21,32,45^. Of note, all the experiments have been performed by using genetic approaches, while no pharmacological modulation of SIRT6 activity have been described. Here we demonstrate that a newly synthesized SIRT6 activator, the pyrrolo[1,2-a]quinoxaline derivative UBCS039, is effective in triggering autophagy in several human cancer cell lines of different histotypes. The results reported here indicate that UBCS039 is specific for SIRT6 deacetylase activity because: (i) UBCS039, but not a chemical analog, increases SIRT6 specific activity on target histone in cells; (ii) UBCS039 is not able to induce autophagy in SIRT6-depleted cells; and (iii) overexpression of wild-type SIRT6, but not of its catalytically inactive mutant, exerts the same effect of UBCS039 on autophagy. UBCS039-specific activity for SIRT6 is further supported by our previous study demonstrating no significant activation of SIRT1, SIRT2, and SIRT3 deacetylase activity by UBCS039 in enzymatic assays^37^. Even though UBCS039 is also able to enhance SIRT5 desuccinylase activity in vitro^37^, pharmacological or genetic inactivation of SIRT5 was reported to increase autophagy^24^, further supporting that the effects of UBCS039 on autophagy is not related to its effect on SIRT5 but are dependent on its ability to activate SIRT6.

Even though our studies reveal for the first time that activation of SIRT6 activity by UBCS039 induces deacetylation of SIRT6-targeted histone H3 sites, we noted difference in UBCS039-mediated H3K9 and H3K56 deacetylation among cell lines. This differential effect may depend on the disparate levels of basal acetylation at H3, as well as on the difference in HAT or HDAC enzymes that have H3K9 or K56 as their substrate.

By using both biochemical and imaging approaches, we reported that the autophagic flux was increased upon UBCS039-mediated SIRT6 activation, as evidenced by canonical conversion of LC3B-I into LC3B-II as well as autophagosome formation and maturation. Digging deeper into the mechanisms by which pharmacological activation of SIRT6 induces autophagy, we found increased intracellular ROS levels, a well-established inducer of the autophagic process^42,43^. Interestingly, antioxidants were able to completely counteract UBCS039-induced autophagy, confirming that ROS burst had a key role in the upstream events leading to autophagy commitment. Even though the molecular mechanisms linking increased ROS production and SIRT6 activation were not investigated in the present work, we can speculate that the increased activity of SIRT6 requires NAD^+^, mainly produced at the level of mitochondrial electron transport chain. The increased ATP level upon SIRT6 activation states for increased NADH oxidation and nicely correlates with increased ROS production. Moreover, it is reported that overexpression of SIRT6 results in increased ROS production and that a specific miR-33a by suppressing SIRT6 induced tumor growth through oxidative stress resistance^46^. Work is in progress in our laboratory in order to fill this gap.

Sirtuins and AMPK are cellular energy sensors and their function and regulation are closely intertwined^1,47^. So far, little is known about the link between SIRT6 and AMPK in cancer. It has been reported that SIRT6 overexpression can activate the AMPK pathway by elevating the AMP/ATP ratio^48,49^. Nevertheless, our results revealed increased ATP levels and AMPK activation upon UBCS039-mediated SIRT6 activation. This apparent contradiction could be explained by the fact that, as already reported^50^, AMPK is a redox sensitive enzyme that is activated by ROS flux. We also demonstrated that UBCS039-mediated SIRT6 activation promotes the formation of phagophores by the activation of AMPK-mTOR-ULK axis. Indeed, UBCS039 decreases mTOR phosphorylation at Ser2448 and increases ULK1 phosphorylation at Ser555, leading to an increase of ULK1-regulated ATG proteins, such as Beclin-1 and Atg5. Nevertheless, we cannot exclude that SIRT6 may also act deacetylating autophagy-related molecules involved in later stages, thus inducing other non-canonical pathway of autophagy. For example, Atg7, Atg8, and Atg12 have been reported to be acetylated and Atg protein acetylation can either promote or inhibit their function in autophagy^28^.

Our study also reports the cellular consequences of autophagy induction by the pharmacological activation of SIRT6, demonstrating that UBCS039 limits the growth of several cancer cell lines by eliciting apoptosis. The crosstalk between autophagy and apoptosis was directly assessed by showing that: (i) the inactive compound UBCS060, unable to both activate SIRT6 and induce autophagy, failed to reduce cancer cell growth; and (ii) the CQ chemical inhibitor of autophagy partially reverts UBCS039 effects on apoptosis. Of note, since the pan-caspase inhibitor zVAD-fmk completely reverts apoptosis induced by UBCS039, we cannot exclude an autophagy-independent UBCS039-mediated cell death.

Autophagy and its relationship with apoptosis in cancer has been widely studied and now it is well known that autophagy can either suppress or activate cell death^34,51,52^. Our results strongly indicate that an excessive stimulation of autophagy can be lethal for cancer cells with different apoptotic thresholds and genetic background.

Overall, our results give a preclinical proof of concept that the direct pharmacological activation of SIRT6 results in autophagy induction, providing a strong rationale for the development of innovative anticancer (combined) therapies based on activation of sirtuins.

## Electronic supplementary material

Supplemental Figures
