# Supplementary material for: Pharmacological activation of SIRT6 triggers lethal autophagy in human cancer cells
Source: Cell Death Dis. 2018 Sep 24;9(10):996. doi: 10.1038/s41419-018-1065-0 (PMC6155207; doi:10.1038/s41419-018-1065-0)
Supplement: Supplementary file 1 — Supplemental Figures [file 41419_2018_1065_MOESM1_ESM.pdf]

Supplementary 1

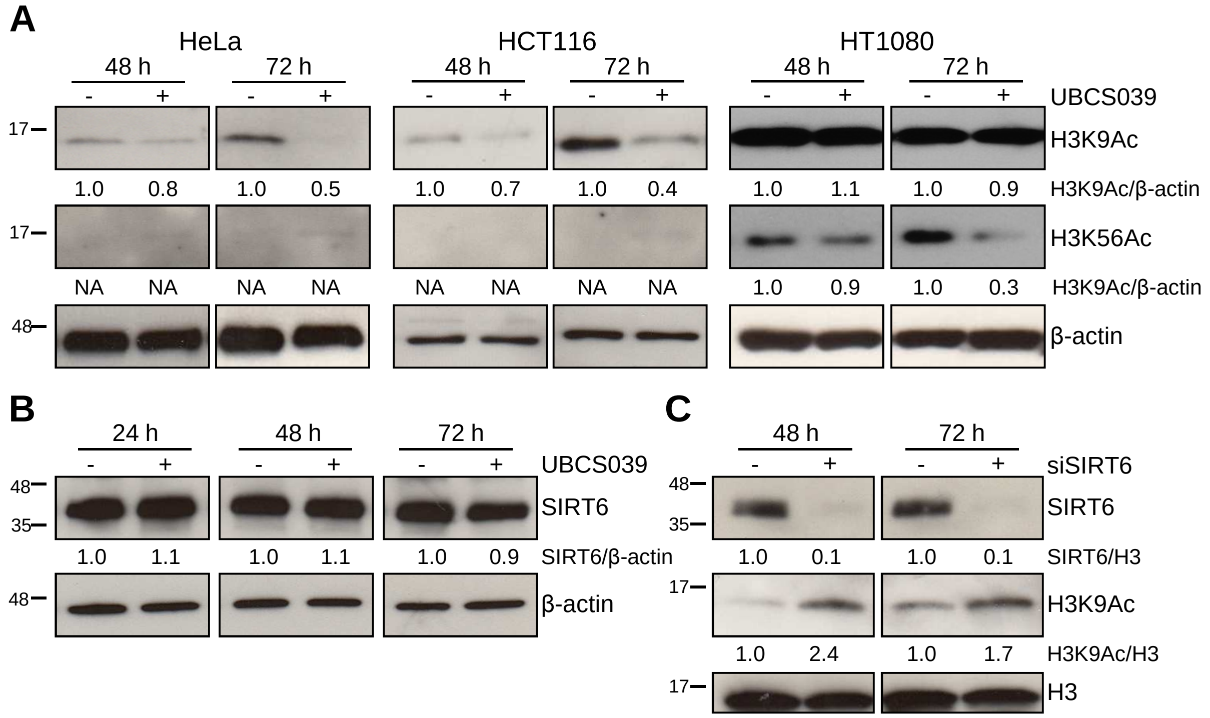

Supplementary 1. In vivo SIRT6 activation by UBCS039 treatment is a general phenomenon.
A) HeLa, HCT116 and HT1080 cancer cell lines were treated with 75 µM UBCS039 for 48 and 72 h. Western blot analysis of acetylation levels of histone H3K9 and H3K56. β-actin was used as an internal loading control. B) Western blot analysis of SIRT6 in HCT116 cancer cells treated with 75 µM UBCS039 for the indicated times. β-actin was used as an internal loading control. C) H1299 cancer cells were transiently interfered for SIRT6 expression for 48 and 72 h and acetylation levels of H3K9 were analyzed. SIRT6 protein levels were used as interference control and H3 total protein levels as an internal loading control. Numbers represent Western Blot quantification by ImageJ software.

Supplementary 2

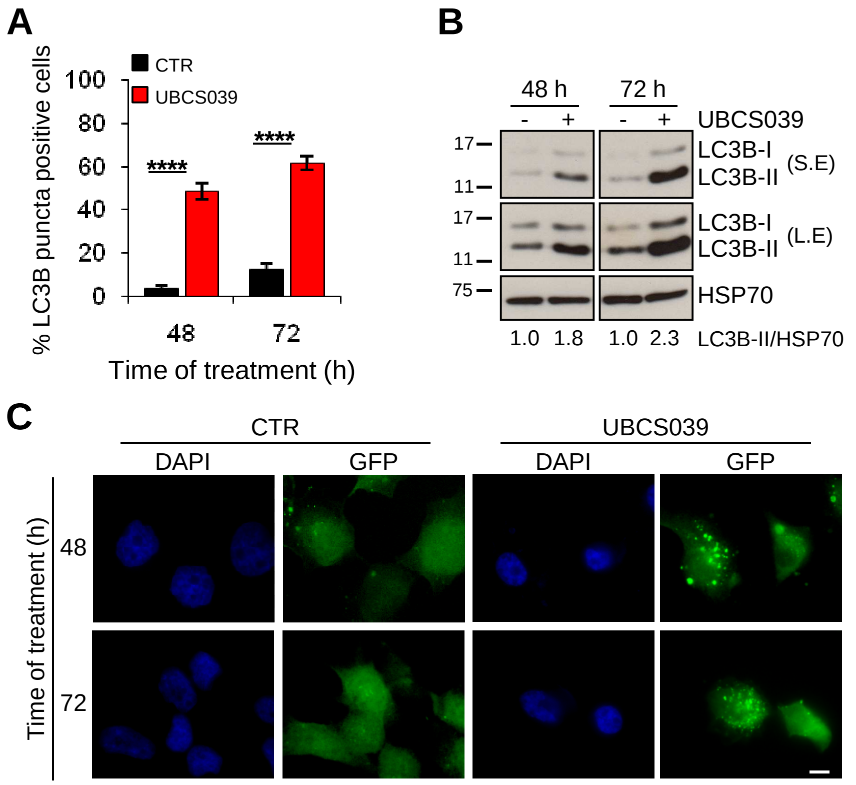

Supplementary 2. UBCS039-induced autophagosomes accumulation is a general phenomenon. HT1080 EGFP-LC3B cancer cells were treated with 75 µM UBCS039 for the indicated times. **A)** Quantitative analysis of LC3B puncta positive cells. The results represent mean ± SEM of three independent experiments. ****P <0.0001. **B)** Western blot analysis of LC3B-II. HSP70 was used as an internal loading control. Numbers represent Western Blot quantification by ImageJ software. S.E. Short exposure; L.E. Long exposure **C)** Representative images of LC3B puncta positive cells (Olympus AX70 microscope , 100 X magnification). Scale bar indicates 10 µm.

Supplementary 3

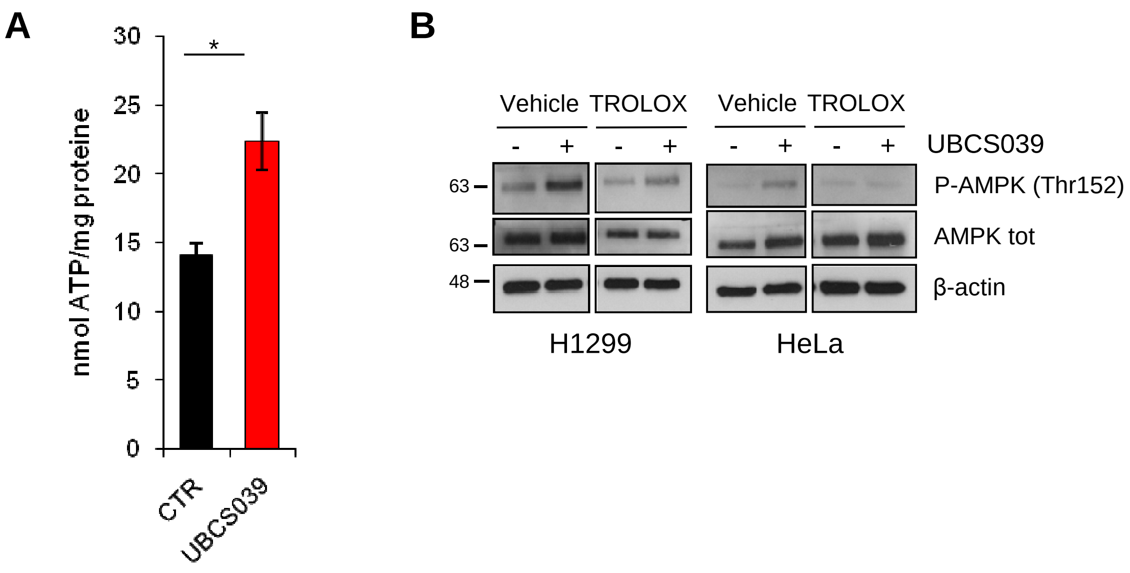

Supplementary 3. Trolox partially reverts the effect of UBCS039 on AMPK pathways.
A) ATP levels evaluation in HeLa cancer cells after 24 h of 100 µM UBCS039 treatment. Results are expressed as mean ± DS of three independent experiments. *P <0.05. **B)** Cells were treated with 100 µM UBCS039 for 48 h in combination or not with anti-oxidant compound 200 µM Trolox. Western blot analysis of the indicate proteins. β-actin was used as internal loading controls.

Supplementary 4

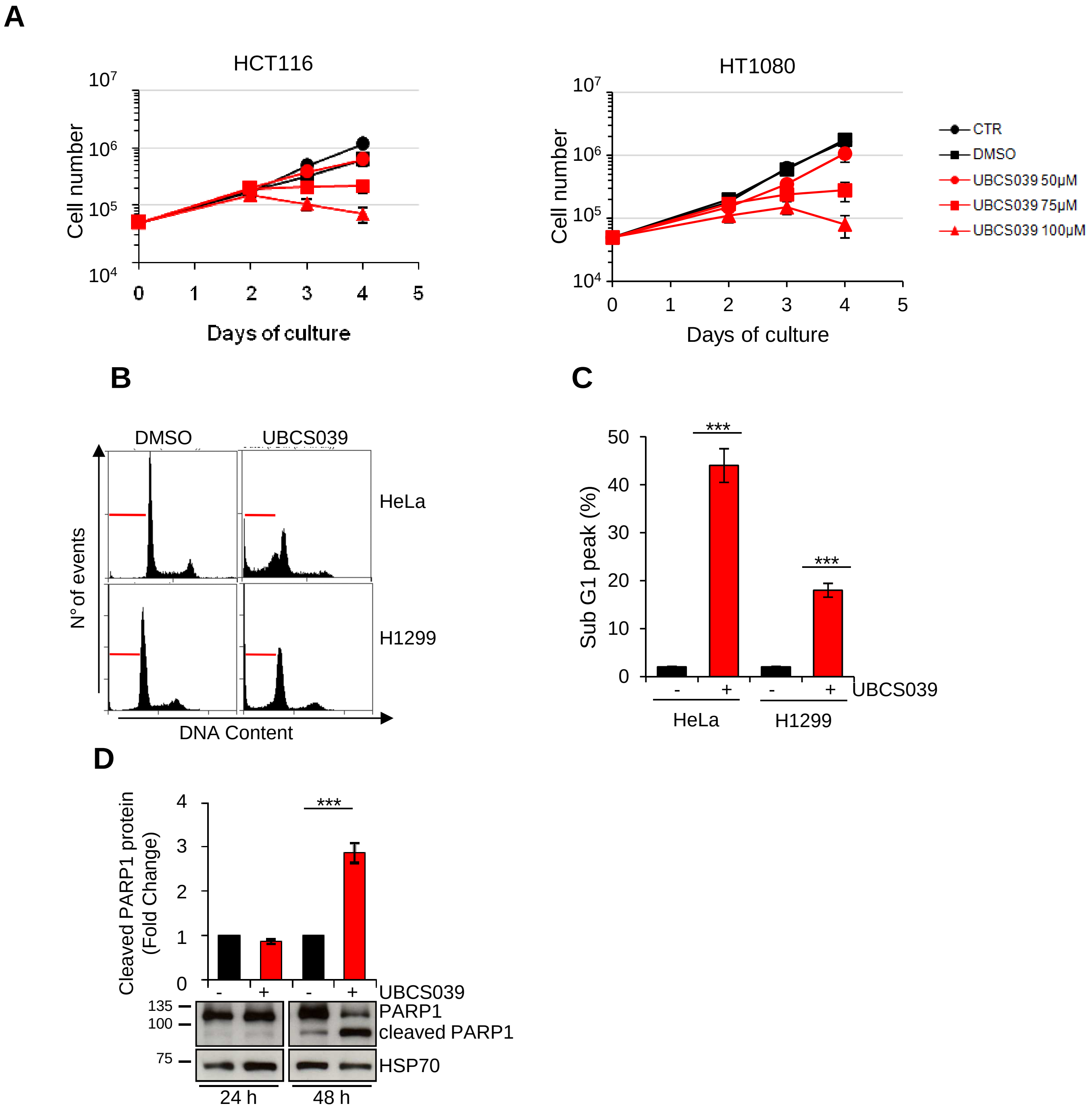

Supplementary 4. UBCS039 treatment impairs cell proliferation.
A) *In vitro* growth curves of HCT116 and HT1080 human cancer cells treated or not with UBCS039 at the indicated doses. $5x10^4$ cells were seeded at day 0 and treated at day 1. The figure represents mean ± DS of three independent experiments. **B,C)** Representative experiment of flow cytometric analysis of AnnexinV–positive cells in HeLa and H1299 human cancer cells treated with 100 µM UBCS039 for 72 h. The results represent mean ± DS of three independent experiments. ***P <0.001 **D)** Western blot analysis of PARP1. Relative protein levels of PARP1 cleaved form were expressed in the histogram as fold changes of treated versus untreated samples, after HSP70 normalization . ***P <0.001
